# Deglacial patterns of South Pacific overturning inferred from ^231^Pa and ^230^Th

**DOI:** 10.1038/s41598-021-00111-1

**Published:** 2021-10-14

**Authors:** Thomas A. Ronge, Jörg Lippold, Walter Geibert, Samuel L. Jaccard, Sebastian Mieruch-Schnülle, Finn Süfke, Ralf Tiedemann

**Affiliations:** 1 Department of Marine Geology, Alfred-Wegener-Institut Helmholtz Zentrum für Polar-und Meeresforschung, PO Box 120161, 27515 Bremerhaven, Germany; 2 Institut für Geowissenschaften, Universität Heidelberg, 69120 Heidelberg, Germany; 3 MARUM Center for Marine Environmental Sciences, Universität Bremen, 28334 Bremen, Germany; 4 Institut für Geologie & Oeschger Center for Climate Change Research, Universität Bern, 3012 Bern, Switzerland; 5 Institute of Earth Sciences, University of Lausanne, 1015 Lausanne, Switzerland

**Keywords:** Biogeochemistry, Palaeoceanography, Palaeoclimate

## Abstract

The millennial-scale variability of the Atlantic Meridional Overturning Circulation (AMOC) is well documented for the last glacial termination and beyond. Despite its importance for the climate system, the evolution of the South Pacific overturning circulation (SPOC) is by far less well understood. A recently published study highlights the potential applicability of the ^231^Pa/^230^Th-proxy in the Pacific. Here, we present five sedimentary down-core profiles of ^231^Pa/^230^Th-ratios measured on a depth transect from the Pacific sector of the Southern Ocean to test this hypothesis using downcore records. Our data are consistent with an increase in SPOC as early as 20 ka that peaked during Heinrich Stadial 1. The timing indicates that the SPOC did not simply react to AMOC changes via the bipolar seesaw but were triggered via Southern Hemisphere processes.

## Introduction

The end of the last glacial was marked by several dramatic changes in the climate system. Global temperatures rose by ~ 4 °C^Ref.^^1^, ice sheets retreated globally^2,3^, while the concentration of atmospheric CO_2_ increased by ~ 90 ppm across the last glacial transition^4^. Several studies imply that changes in the strength and geometry of the Atlantic Meridional Overturning Circulation (AMOC) coincided with transient climate perturbations, punctuating the deglacial warming^5^. Concomitant with the Northern Hemisphere (NH) cold intervals, Heinrich Stadial 1 (HS1; 17.5–14.7 ka) and the Younger Dryas (YD; 12.9–11.7 ka), the AMOC weakened/shoaled in response to changes in buoyancy forcing^5^. Simultaneously, the Southern Hemisphere (SH) experienced several—antiphased—modifications in the ocean–atmosphere system^6^.

Thus far, the formation and dynamics of North Atlantic Deep Water (NADW) and more broadly the AMOC were in the focus of studies reconstructing past changes in the global overturning circulation. Notwithstanding the importance of NADW-formation for the ventilation of the oceans subsurface as a major contributor to the global Thermohaline Circulation (THC), it is equally crucial to understand the evolution of its main counterpart, the Southern Ocean (SO), where bottom- and intermediate-waters are formed and advected to all ocean basins. Anderson et al.^7^ showed that SO upwelling significantly increased ~ 18,000 years before present (ka) at the beginning of the last glacial termination. Collapsing Antarctic ice sheets^2,3^ and rising atmospheric CO_2_^Ref.^^4^ accompanied enhanced upwelling south of the Antarctic Polar Front, highlighting the importance of Southern Ocean overturning in propelling the global climate system out of the last glacial^8–12^. In the Pacific sector of the SO however, our knowledge related to circulation patterns on glacial-interglacial and millennial timescales, remains fragmentary.

Modeling studies suggested that, depending on boundary conditions, changes in the AMOC may directly impact Pacific overturning via atmospheric teleconnections^13^, raising the following questions: (i) can the bipolar seesaw hypothesis^14^, based on Atlantic data also explain the reconstructed deglacial changes in the Pacific overturning circulation in general, and the South Pacific overturning circulation (SPOC) specifically? (ii) did the SPOC react to changes in AMOC, or did it evolve independently?

**Figure 1:**
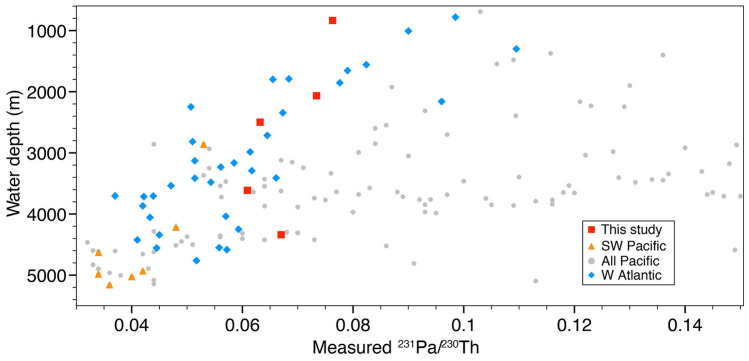
Holocene ^231^Pa/^230^Th from the Pacific (grey dots)^15^, the SW-Pacific (orange triangles^15^; red squares, this study), and the W Atlantic (blue diamonds; Table S1). Decreasing ^231^Pa/^230^Th with water depth in the W Atlantic (blue) is interpreted as an imprint of AMOC, while in the Pacific (grey) a similar correlation is observed only for the SW region (orang, red).

**Figure 2:**
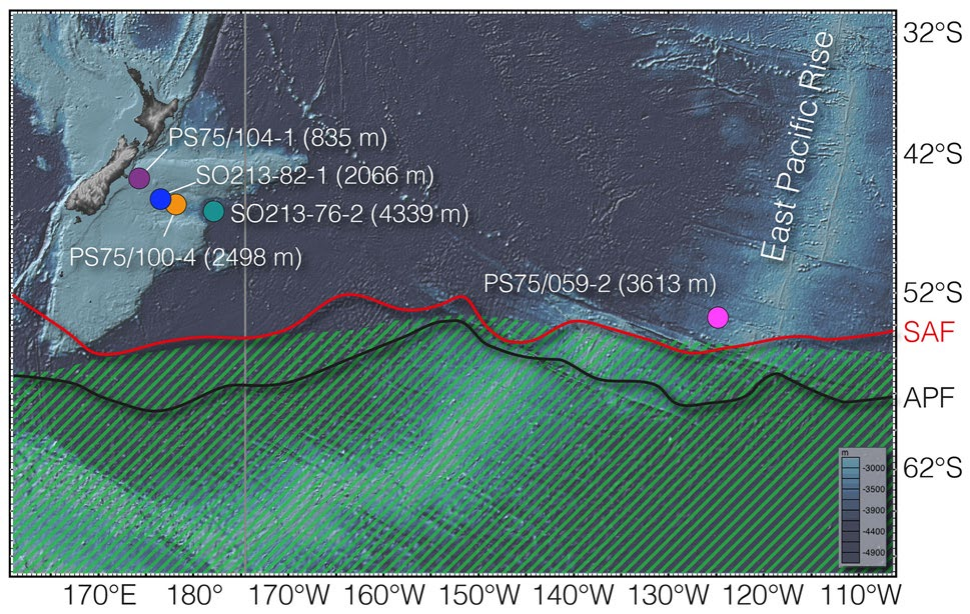
Research area. Colored dots—core locations; SAF—Subtropical Front (red line); APF—Antarctic Polar Front (black line)^68^; Green shading—opal belt. Vertical grey line—section used in Fig. 6. Map created with GeoMapApp 3.6.12 (http://www.geomapapp.org).

Luo et al.^15^ analyzed the influence of deep-water circulation on the spatial distribution of ^231^Pa and ^230^Th in the Pacific Ocean (Fig. 1). Based on their findings, we aim to test the applicability of ^231^Pa/^230^Th as a paleo circulation proxy in the SW-Pacific (Fig. 2), in the context of gaining further insights into the questions outlined above.

Generally, Pacific seawater ^231^Pa/^230^Th-ratios are modulated by the longer residence time of deep-waters and thus reflect higher boundary scavenging intensity at the continental margins when compared to the Atlantic basin^16–18^. Accordingly, ^231^Pa/^230^Th has yet mainly been applied to reconstruct spatio-temporal changes in the MOC regimes of Atlantic and not in the Pacific basins. An active meridional overturning cell induces a general decrease in sedimentary ^231^Pa/^230^Th with water-depth as observed in the Atlantic Ocean^19–21^. The reported depth-dependent decrease is a consequence of the difference in particle reactivity between the two radionuclides with the relatively less particle-reactive ^231^Pa, which is more prone to be advected, while ^230^Th is preferentially exported and deposited into the underlying sediments, by reversible particle scavenging. Recently Luo et al.^15^ examined to which extent the manifestation of ocean circulation can be recorded in core top sediments based on a compilation of > 250 ^231^Pa/^230^Th measurements, covering large swaths of the Pacific Ocean (Fig. 1). The basin wide data-distribution underlines the anticipated predominant influence of particle fluxes and boundary scavenging sedimentary ^231^Pa/^230^Th. Yet, regional subdivisions of the data set reveal that in the central gyres and the Southwest Pacific region a discernable influence of SPOC is recorded by sedimentary ^231^Pa/^230^Th values. The vertical attenuation in ^231^Pa/^230^Th values along water depth in these regions, similar to the Atlantic, is interpreted as indicative of the influence of the overturning circulation^15^. Building on these findings, we measured ^231^Pa/^230^Th downcore profiles based on five sediment cores retrieved from the SW Pacific along a depth transect ranging between 835 and 4339 m back to ~ 30 ka to provide evidence for glacial and deglacial variations in PMOC dynamics.

## Results

Here we reconstruct changes in the SPOC from a depth transect of five sediment cores from the New Zealand Margin and the East Pacific Rise (Fig. 2). These sediment cores are bathed by the major Southwest Pacific deep-water masses, including Antarctic Intermediate Water (AAIW) the Upper and Lower Circumpolar Deep Water (UCDW and LCDW) as well as the Antarctic Bottom Water (AABW).

**Figure 3:**
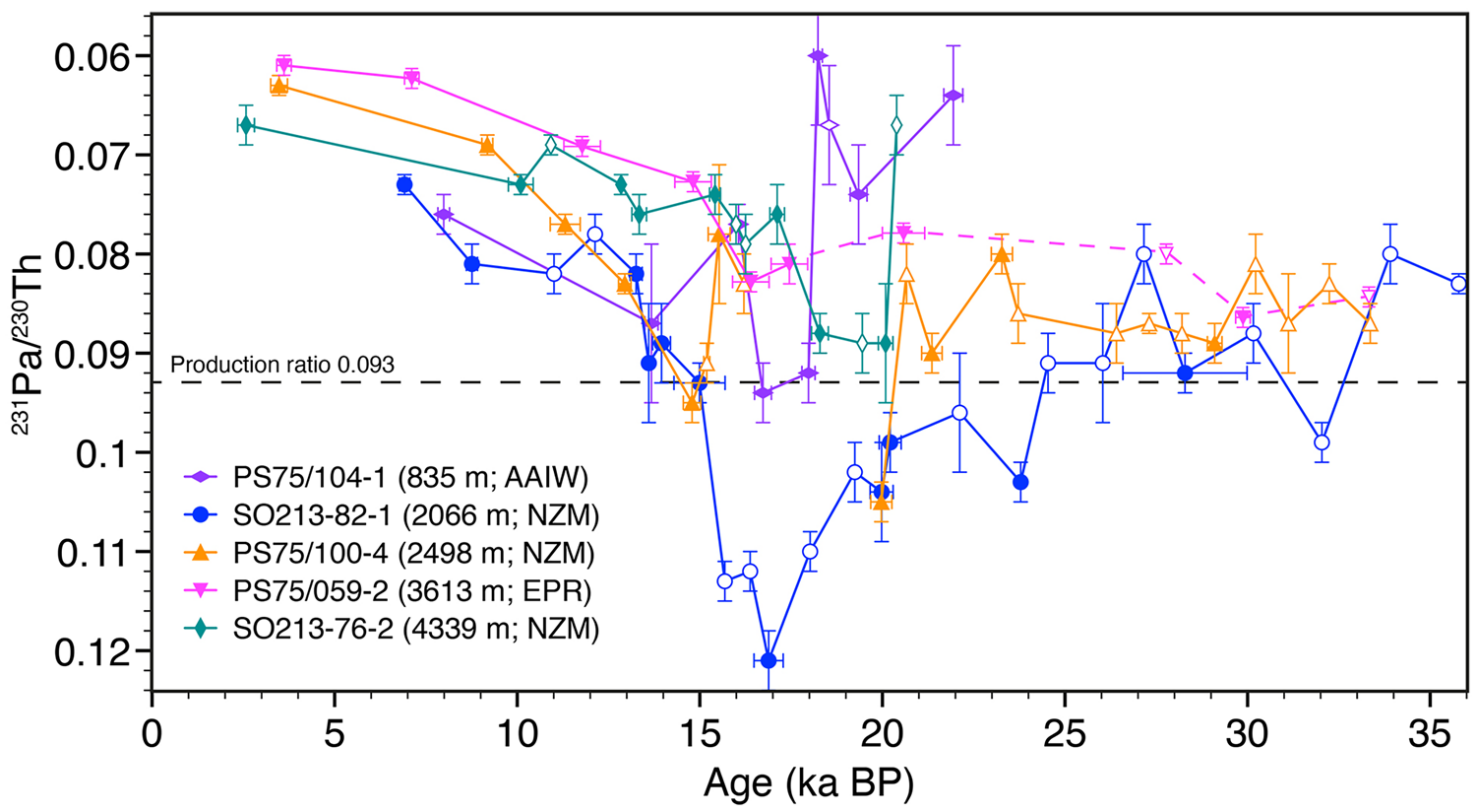
Patterns of deep-water ^231^Pa/^230^Th. Dashed pink line indicates time interval when influences by the advancing glacial opal belt cannot be ruled out (supplementary information). Filled symbols—samples with parallel ^14^C measurements. Please note that ^14^C-Age error bars are sometimes smaller than the symbols used.

**Figure 4:**
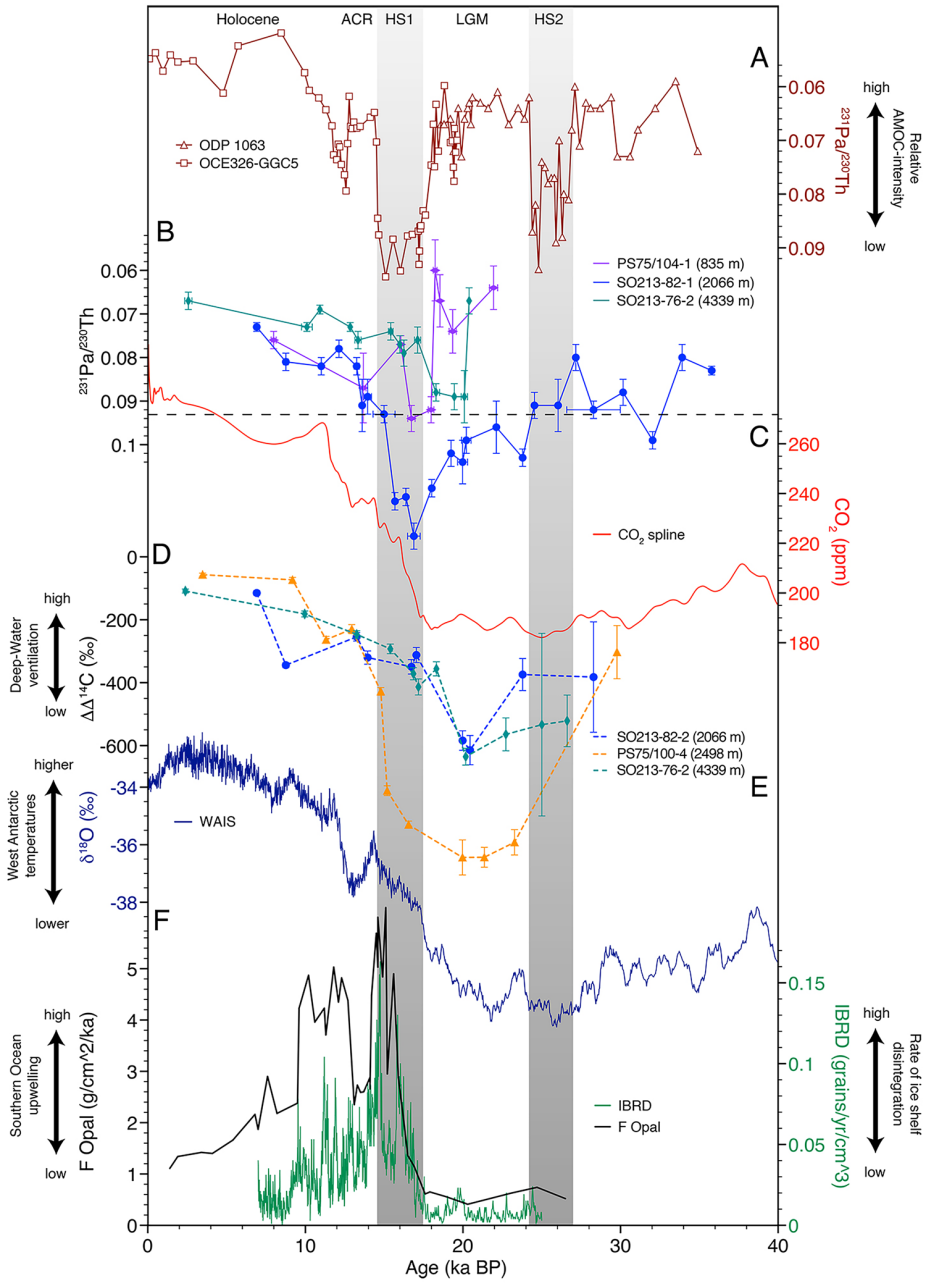
Evolution of glacial-interglacial ocean–atmosphere patterns (**A**) North Atlantic records (brown) OCE326-GGC5^Ref.^^5^ and ODP 1063^Ref.^^28^. (**B**) Southwest Pacific ^231^Pa/^230^Th records (this study). The dashed line indicates the ^231^Pa/^230^Th production ratio. (**C**) Atmospheric CO_2_—red^69^. (**D**) Deep-water ΔΔ^14^C^Ref.^^10^. (**E**) Smoothed West Antarctic δ^18^O^Ref.^^42^. (**F**) Scotia Sea Iceberg rafted debris (IBRD)—green^3^; South Atlantic opal flux—black^7^. ACR—Antarctic Cold Reversal; HS—Heinrich Stadial; LGM—Last Glacial Maximum.

**Figure 5:**
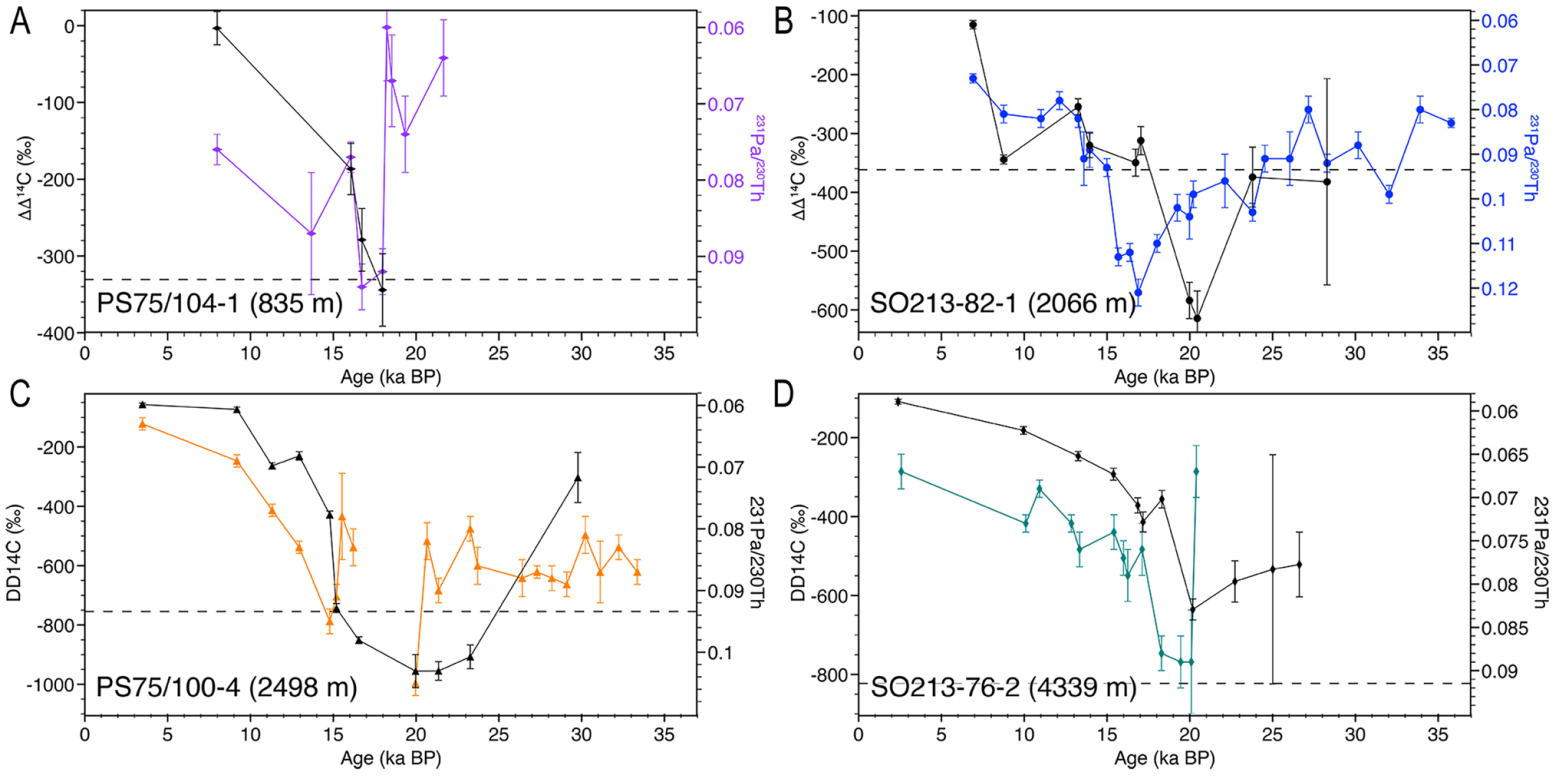
Comparison of ^231^Pa/^230^Th-data (this study) to published radiocarbon records, measured on the—same sediment cores^10,56^. Colors as indicated in Fig. 1. Black lines—ΔΔ^14^C; Please note that some additional ^14^C-data were added for SO213-82-1 (**A**) as indicated in Table S2. The dashed lines indicate the ^231^Pa/^230^Th production ratio.

To assess temporal variations in the dynamic of SPOC, we make use of the sedimentary ratio of ^231^Pa and ^230^Th, which based on the recent results by Luo et al.^15^ (Fig. 1), suggest that the Southwest Pacific is an area sensitive to circulation driven changes in ^231^Pa/^230^Th, as well as published ΔΔ^14^C-records^10^ (Figs. 3, 4, 5). In the open ocean, the residence time of ^231^Pa is about 10-times higher than of ^230^Th^Ref.^^22^. Oceanic circulation thus results in the enhanced advection of ^231^Pa and hence ^231^Pa/^230^Th values below the production ratio (0.093)^23^. Consequently, if deep-water circulation weakens, sedimentary ^231^Pa/^230^Th increases toward the production ratio^21,24^. As biogenic opal is widely known to decrease the residence-time of ^231^Pa by preferentially removing it from the water column^25^, we analyzed the opal contents along with ^231^Pa/^230^Th (PANGAEA). The amount of biogenic opal in most of our samples is very low (< 3 wt%) and—more importantly—does not correlate with the pattern of ^231^Pa/^230^Th, implying that a significant impact on the scavenging behavior of ^231^Pa at our core locations remains improbable^26^. However, we consider the contribution of biogenic opal export production poleward (i.e. upstream) of the core locations on the local ^231^Pa/^230^Th signal (supplementary information).

Reaching back ~ 35,000 years, our dataset displays coherent variations in the sedimentary ^231^Pa/^230^Th values. The general trends of the independent ^231^Pa/^230^Th-data and ΔΔ^14^C-records^10^, with bulges in the records in the glacial and deglacial sections imply a certain level of consistency throughout time and space (Fig. 4). Glacial ^231^Pa/^230^Th-ratios are generally higher and more variable than during the Holocene (Fig. 3), as are the ΔΔ^14^C-records of this region^10^. The most noticeable feature is a transient increase in ^231^Pa/^230^Th values between ~ 20 and 18 ka in cores PS75/104-1, SO213-76-2, and SO213-82-1. With a maximum of 0.12, the signal recorded by SO213-82-1 is particularly salient, as it significantly exceeds the production ratio. After ~ 17 ka, we report a constant decrease in ^231^Pa/^230^Th values gradually declining toward Holocene values in all sediment records (Fig. 3).

## Discussion

The bathymetric transect of sediment cores presented here comprise records from different water depths and thus, different water masses and circulation regimes. The shallowest record PS75/104-1, is bathed by AAIW recently formed in the Antarctic Polar Zone, an area of high opal production (Fig. 1). This implies that changes in opal export production, upstream of our core location might have a significant impact on the ^231^Pa/^230^Th pattern locally^27^. Thus, we interpret this record as well as the older part of PS75/059-2 (> 18 ka), when the main area of high opal production migrated northwards, reflecting a combination of opal productivity, and (to a lesser extent) ocean circulation (see supplementary information for more details, Fig. S1). In a similar way to PS75/104-1, AABW record SO213-76-2 might have been influenced by waters recently formed in an area of high opal production (Fig. S1). However, as this core location is also under the influence of an admixture of LCDW, we assume that this effect is less severe than in our AAIW record. Mid-depth records SO213-82-1 and PS75/100-4 are bathed by southbound CDW/PDW (Circumpolar/Pacific Deep Water). It is expected that the concentration of ^231^Pa builds up relatively to ^230^Th along increasing travel time as a function of PMOC strength. Thus, changes in the upstream export of ^231^Pa are able to exert a dominant influence on the SW-Pacific ^231^Pa/^230^Th ratios as shown by Luo et al.^15^. Holocene ratios of all sediment cores (Fig. 3) fall within the modern budget of the SW-Pacific^17^, ranging between ~ 0.06 and 0.08.

During the last glacial, the deep-water cores indicate low ΔΔ^14^C-values^10^ and ^231^Pa/^230^Th-values close to or even higher than the production ratio. In general, we observe generally higher ^231^Pa/^230^Th-ratios during the glacial, compared to the Holocene, indicative of either weaker SPOC, higher glacial particle fluxes or most likely a combination of both (Fig. 1)^15^.

The deglacial trends of the mid-depth ^231^Pa/^230^Th-profiles (SO213-82-1 and PS75/100-4) are reminiscent of the North Atlantic ^231^Pa/^230^Th-records from the Bermuda Rise^5,28^. The Southwest ^231^Pa/^230^Th patterns are in good agreement with other studies reconstructing circulation and ventilation in the Pacific^10,29–32^, Drake Passage^33^, South Atlantic^8^, and South Indian Ocean^34^.

The comparison of ^231^Pa/^230^Th from the CDW cores (SO213-82-1 and PS75/100-4) and Atlantic ^231^Pa/^230^Th, reveals certain similarities but also striking differences on millennial-timescales. The Bermuda Rise records^5,28^ show prominent phases of an increased ^231^Pa/^230^Th during HS2, HS1 and YD, interrupting the generally constant Glacial and Holocene ^231^Pa/^230^Th-baseline (Fig. 4A). Our Southwest Pacific mid-depth records however, show an evolution from more variable, high glacial to gradually decreasing lower Holocene values (Fig. 4B), interrupted by a transient event of elevated ^231^Pa/^230^Th-values (~ 20–14 ka). In order to investigate if this general glacial to Holocene trend reflects a glacial reduction in upstream removal of ^231^Pa, we turned to several sediment records along the Equatorial East Pacific Rise. These records show a consistent trend from lower glacial, to increased Holocene values that approach and exceed the production ratio between ~ 17 ka and 11 ka (Fig. S2)^35^. It is thus plausible that the decrease in ^231^Pa/^230^Th as observed in our records (Fig. 3), represents diminished export of ^231^Pa from the north after ~ 17 ka. It is plausible to assume that the equatorial records^35^ were themselves also influenced by upstream processes, which is in good agreement with the processes outlined by Luo et al.^15^. Nevertheless, it is important to note, that these records are probably not exactly upstream of our core locations but provide the only available approximation of an upstream signal from ^231^Pa/^230^Th downcore profiles from the literature to date.

**Figure 6:**
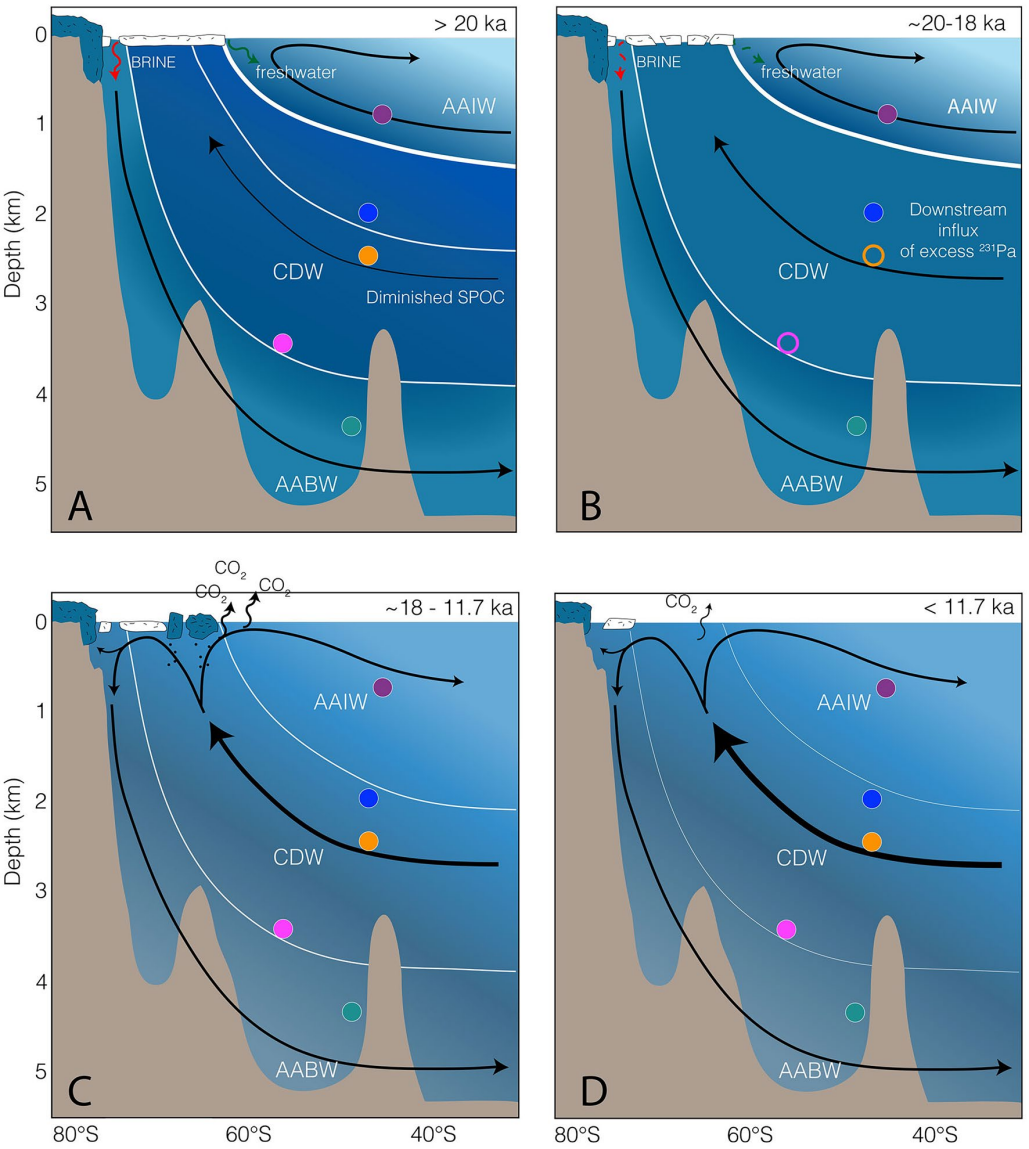
South Pacific overturning and ventilation. (**A**) Last Glacial. Diminished SPOC with separated deep- and intermediate-water cells. SO213-76-2 (green dot) under the influence of LCDW and AABW (**B**) LGM. Increasing SPOC transports excess ^231^Pa downstream toward the CDW core-locations. (**C**) Deglacial Transition. Progressive increase in SPOC results in the release of CO_2_ and the transport of warm deep-waters onto the shelf regions. Influx of excess ^231^Pa from the upstream regions diminishes. Disintegration of ice-shelves and release of Iceberg Rafted Debris (black dots). (**D**) Holocene pattern of the SPOC. Colored dots—sediment records as shown in Fig. 2. Empty circles—no data in this time slice. White lines—assumed isopycnals, the thicker the lines, the stronger the vertical stratification. Black arrows—water mass circulation, thickness of the central arrow indicates relative SPOC strength. Color shading of the CDW-cell indicates its ventilation state according to Ronge et al. (2016). Dark blue—high CO_2_, low ΔΔ^14^C.

Despite a sufficient temporal resolution, we did not observe any changes during HS2 as manifested in Atlantic sediment cores (Fig. 3). Depending on the boundary conditions, HS2 might not have been associated with any sizeable variation in the SPOC^13^. An additional feature of our records is the inverse evolution of ^231^Pa/^230^Th and ΔΔ^14^C throughout the end of the last glacial (Figs. 3, 4, 5). At ~ 20 ka, our data show increased ^231^Pa/^230^Th-ratios, accompanied by enhanced ventilation (ΔΔ^14^C)^10^ (Figs. 4, 5). Appearing contradictory at first, we argue that both patterns are likely the result of increasing water mass advection. Upon SPOC increase, excess ^231^Pa gets transported via PDW/CDW downstream to our core locations^15^, increasing the ^231^Pa/^230^Th-ratio far above the production ratio (Figs. 4, 6B).

Hence, at this water-depth, increased ^231^Pa/^230^Th-values may not reflect a SPOC slow-down^15^ but rather imply its reinvigoration. This is in good agreement with the ΔΔ^14^C-records that indicate more active overturning and ventilation during this time period^10^. Thus, the evolution of both proxies (^231^Pa/^230^Th and ΔΔ^14^C) can be explained with the same mechanism, an increase in SPOC that transported excess ^231^Pa to our core location, while also leading to an increase in ΔΔ^14^C^Ref.^^10^. From ~ 19 ka on, PS75/104-1 (AAIW) recorded lower ^231^Pa/^230^Th-ratios, while the mid-depth cores still experienced elevated values (Fig. 3). This short interval was likely caused by increasing opal production in the Antarctic Zone of the Southern Ocean^7^ that stripped ^231^Pa from the water before reaching the downstream location. Subsequent to this peak, ^231^Pa/^230^Th-values rapidly increased and paralleled the pattern observed in both mid-depth cores (Fig. 3). Following this drastic rise in ^231^Pa/^230^Th, we observed decreasing values during HS1 (PS75/104-1; PS75/100-4; SO213-82-1; Figs. 3, 4). With the continuous deglacial SPOC-strengthening, the supply of excess ^231^Pa^Ref.^^15^ progressively abated, so that ^231^Pa/^230^Th-ratios approached modern-like values toward the Holocene.

While the AMOC plummeted, South Pacific ^231^Pa/^230^Th- and ΔΔ^14^C-data (Fig. 4) indicate a progressive evolution of the SPOC toward modern values, starting as early as ~ 20 ka, which culminated during HS1 (Fig. 4). Other records from the Southwest Pacific also corroborate this timing, featuring a significant decrease of εNd as a result of deep-water destratification and enhanced mixing^11^.

The significantly different patterns of AMOC vs. SPOC bring us back to our initial questions. Did NH changes force the South Pacific via bipolar teleconnections, or were climatic changes on the SH the driving factors?

The declining ventilation and circulation of mid-depth SO waters^8,10,29^ paralleled peak glacial SH climate such as low temperatures, changes in the density structure of intermediate- and bottom-waters, expanded sea ice, and displaced or weakened Southern Westerly Winds (SWW)^36–39^. According to the age models of our sediment cores, the mid-depth SPOC recovered faster from the HS1 disturbance than the AMOC (Fig. 4). Hence, the initial impulse that triggered the increase in deglacial SPOC and upwelling^7^ must have arisen from the SO or the SH and not via NADW. During this period, glacial climate conditions reversed and gradually exposed the upwelling area of CDW to the surface. The combination of different parameters such as reduced northward heat advection and shifts in the Intertropical Convergence Zone and wind belts increased Southern Ocean upwelling and CO_2_-release^40,41^. In addition, local changes in orbital forcing, are considered to be an important factor driving early deglacial changes in the West Antarctic and Pacific sector^40^. Hence, we argue that the SPOC increase that preceded the end of the LGM (Fig. 6A) was triggered by processes centered in the SH and not by changes in NADW dynamics. In this respect, independent records of Antarctic Ice Sheet retreat^3^ and the early West Antarctic warming phase^40,42^ are consistent with the timing observed in our records (Fig. 4).

Deep- to bottom-water sediment core SO213-76-2 (4339 m) however, differs from the deglacial pattern of the other records, as it marks an interval of decreasing ^231^Pa/^230^Th from ~ 19 on (Figs. 4B, 5). This interval is in very good agreement to the increasing ΔΔ^14^C-values measured on the same sediment core^10^ that also show a similar shift at ~ 20 ka (Fig. 5). Today, SO213-76-2 is influenced by LCDW and AABW^29^. During the LGM however, the core location of SO213-76-2 was probably only exposed to LCDW. We argue that during peak glacial times, AABW was too dense to be exported to the north of the Pacific Antarctic Ridge, in a similar manner as it was observed in the Atlantic sector of the SO^43^. In the Pacific sector, processes associated with the early Southern Hemisphere warming^42^ began to erode the deep stratification at ~ 20ka^11^. This erosion allowed AABW to reach the core location and thus reduce the influence of excess CDW ^231^Pa on SO212-76-2.

With our new data, we might also be able to add to a debate of Pacific studies that argued for or against a glacial reduction in Pacific overturning^11,44–47^. Our results are consistent with the notion of enhanced glacial carbon storage within mid-depth South Pacific deep-waters^10,45^. In combination with more complete surface nutrient consumption^36,48^ the reported slowdown in glacial SPOC might account for the sequestration of carbon in the deep sea along with a progressive decay of its ^14^C-content. However, to confine the sequestered carbon into the deeper ocean, climatic conditions must have changed in parallel. The stratification of the Southern Ocean’s water column was intensified by an increased glacial density gradient^11,37,39^, Antarctic sea ice noticeably expanded to the north^49^, while the SWW were displaced toward the north^38^. Ultimately, all factors significantly hampered the exchange of deep-waters and the atmosphere. In combination, stratification, expanding sea ice, and displaced winds reduced the upwelling of Circumpolar Deep Waters (CDW) during the glacial, thus are likely the driving parameters behind the observed slowdown of the SPOC. This glacial slowdown extended the residence time of deep-waters within the ocean’s interior by several thousand years^8,10,33,34^, increased ^231^Pa/^230^Th-ratios due to boundary scavenging^15^, and ultimately allowed for the progressive accumulation of carbon (CO_2_) in the glacial ocean.

At the final phase of the LGM, we argue that South Pacific overturning progressively increased (Fig. 6). Possibly supported by atmospheric teleconnections, the increase in SPOC sparked the re-ventilation of Southwest Pacific deep-waters, leading North Atlantic processes by almost 2000 years (Fig. 4). This mechanism transported excess ^231^Pa toward the South Pacific, resulting in the observed transient increase in ^231^Pa/^230^Th. Ultimately, this process is probably linked to the upwelling of carbon-rich deep-water and culminated in the transfer of old, ^14^C-depleted CO_2_ to the atmosphere (Fig. 6C). In addition, the re-invigoration of the SPOC is also a likely process, which carried warm CDW onto the shelf regions, fostered the early retreat of Antarctic ice shelves^2,3^ (Figs. 4B,F, 6C). Hence, our reconstructions of South Pacific overturning present a physical link between increasing deep-water ΔΔ^14^C^Refs.^^8,10,33,34^, declining atmospheric Δ^14^C^Ref.^^50^, rising atmospheric CO_2_-levels^4^, retreating Antarctic ice shelves^3^, and rising global sea level. However, as mentioned above, several reconstructions of benthic δ^13^C values and sortable silt^44^ and foraminiferal εNd^Ref.^^47^ argue against a pronounced decrease in glacial deep Pacific overturning. To a large extent, these studies cover water masses deeper than 3000 m. As our main signal was observed in a water depth of 2066 m, that is also in the range of the so-called floating glacial carbon pool^10,51^, this discrepancy might be explained by the different hydrographic sections sampled. However, to obtain a more comprehensive overview in past SPOC changes and ^231^Pa/^230^Th budgets, we stress the need for more Pacific downcore records.

## Conclusions

Our analysis of downcore ^231^Pa/^230^Th-records based on SW-Pacific sediment cores allowed us to investigate the applicability of this proxy with respect to Pacific core top ^231^Pa/^230^Th-data^15^. In addition, when interpreted as a circulation signal, our new ^231^Pa/^230^Th data set sheds light on the impact changes in the SPOC had on the glacial Pacific carbon pool, and it’s deglacial erosion, and the release of CO_2_. In particular, we conclude that:

1. Following the transient maximum of excess ^231^Pa from the north, all records show a decrease toward Holocene values.
2. Mid-depth Holocene values are considerably lower than glacial values, and are indicative of a strengthened SPOC, compared to the glacial.
3. The ^231^Pa/^230^Th-proxy can be a suitable tool for the investigation of paleo-circulation in the SW-Pacific, if downstream transport of ^231^Pa is accounted for.
4. The hypothetical glacial SPOC reduction would have resulted in a relative increase in ^231^Pa/^230^Th-values in the SW-Pacific off New Zealand, and probably along the EPR compared to the Holocene.
5. With the SPOC reinvigoration at ~ 19 ka, all cores display a significant departure from glacial ^231^Pa/^230^Th-values toward higher levels.
  - As shown in the study by Luo et al.^15^, SW-Pacific ^231^Pa/^230^Th is potentially sensitive to changes in Pacific overturning circulation.
  - Based on this sensitivity, we argue that the deglacial onset of the SPOC presumably transported excess ^231^Pa from the north Pacific to our core locations via PDW/CDW, resulting in values exceeding the production ratio.
6. After ~ 17 ka, upstream records along the Equatorial EPR show a progressive removal of ^231^Pa. This upstream removal likely put an end to the transport of excess ^231^Pa to our cores, causing the progressive decrease in ^231^Pa/^230^Th as observed by us following this time interval.
7. The transient rise in ^231^Pa/^230^Th, during HS, ending earlier in the Pacific than in Atlantic records, is likely indicative of the transport of ^14^C-depleted and CO_2_-rich waters from the floating Pacific carbon pool^10^ toward the surface.
8. Ultimately, this process is a likely common driver between the rise of atmospheric CO_2_^Ref.^^4^, it’s drop in Δ^14^C^Ref.^^50^, and the observed collapse of Antarctic glaciers^3^.
9. Changes in the SPOC did not simply react to AMOC changes via the bipolar seesaw, but were probably triggered by Southern Hemisphere changes in orbital forcing, shifting atmospheric systems, and CO_2_-release^40,41^.
10. Our findings highlight the need for additional downcore records from the South Pacific to obtain a more reliable ^231^Pa/^230^Th as well as better insight into past changes in SPOC.

## Methods

### Sediments and sample treatment

The sediment cores analyzed in our study were recovered during R/V *Polarstern* expedition ANT-XXVI/2 (PS75) in 2010 and R/V *Sonne* expedition SO213/2 in 2011 (Fig. 2). The sediments predominantly consist of foraminifer- and nannofossil-bearing muds with negligible quantities of biogenic opal^52,53^. Sedimentation rates vary between 3.5 and 22.5 cm ka^−1Ref.^^10^. Due to topographic constraints of the Chatham Rise and the East Pacific Rise, the core locations are not affected by changes in the position of the Subtropical and/or Subantarctic fronts^54,55^. The sediment cores form a bathymetric transect that covers the major Southwest Pacific water masses including Antarctic Intermediate Water (PS75/104-1, 835 m), Upper (SO213-82-1, 2066 m; PS75/100-4, 2489 m) and Lower Circumpolar Deep Water (PS75/059-2, 3616 m; SO213-76-2, 4339 m). For the geochemical analyses, we used three to four cubic centimeters of bulk sediment from the working halves, except for core PS75/100-4, where we had to sample the archive halves, as no material was left in the working halves. Unfortunately, PS75/100-4 was so intensively sampled in the deglacial interval that literally no material was left for our analyses of this time period.

### Radiocarbon dating and age models

To better constrain the distinct HS1 pattern of SO213-82-1, we expanded the radiocarbon record of Ronge et al.^10^. Briefly, monospecific planktic foraminifera *Globigerina bulloides* and mixed benthic species (*Cibicidoides* spp. and *Uvigerina* spp), were separated and analyzed at the Alfred-Wegener-Institute’s MICADAS facility. Following the procedure previously outlined in Ronge et al.^10^, we added six data-points into the stratigraphy of core SO213-82-1 and updated the stratigraphy of PS75/104-1 according to Küssner et al.^56^.

However, as the stratigraphy of SO213-82-1 in Ronge et al.^10^ is based on a ^14^C-independent method, via the correlation to the EDC ice core record, we updated these age models using a ^14^C-related approach. To convert ^14^C-ages into calendar ages, we used the Calib 7.1 solution^57^ along with the IntCal13 calibration curve^50^ as well as the New Zealand Margin surface reservoir ages of Skinner et al.^45^. However, as the surface reservoir age reconstruction of Skinner et al.^45^ is insufficient to calibrate all our data points, we also used modelled ^14^C-ages^58^. A direct comparison of modelled^58^ and reconstructed^45^ surface reservoir ages reveals an offset between both methods, ranging from only 20 to about 300 years. To account for the ages of data points without a direct ^14^C-age, we applied two independent Bayesian approaches (see below). Using both methods, we are confident that our age models provide the necessary resolution to discern and characterize millennial-scale changes in deep ocean circulation.

Two samples with prominent planktic age reversals (46–47 cm and 86–87 cm) were ignored for our age models.

Updating the age models resulted only in minor changes in the radiocarbon records provided by Ronge et al.^10^. A comparison of these records to our updated age models is shown in Figure S2. The inclusion of new ΔΔ^14^C data points furthermore improves the agreement of SO213-82-1 with other CDW records from the Pacific (MD97-2121)^45^ and the Atlantic (MD07-3076)^8^.

### Bayesian age modelling

We applied the Bayesian age-depth model *hummingage*, which is developed at AWI and freely available at https://github.com/hummingbird-dev/hummingage. The GitHub repository provides source code for the R programming language and a Jupyter Notebook containing detailed explanations. Additionally, an easy-to-use web service is provided at https://hummingage.awi.de/ for applying *hummingage* online in the browser.

The age-depth model *hummingage* can be an alternative to the widely used *Bacon*^59^ method. In addition to *hummingage* we also applied *Bacon* to our data and show the comparison of both approaches in the supplements (supplementary information; Fig. S4).

### Geochemistry

#### ^231^Pa/^230^Th measurements

The concentrations of sedimentary ^231^Pa, ^238^U, ^230^Th, and ^232^Th were jointly measured by isotope dilution in a co-operation between AWI and Heidelberg University with contributions from the GEOMAR Kiel and using mass spectrometers at the AWI (Element 2), Heidelberg (Element 2, Neptune, iCap) and Kiel (Neptune).

The chemical separation and cleaning followed standard procedures^60^. The ^233^ Pa spike, milked from ^237^Np^Ref.^^61^ was calibrated against the reference standard material UREM-11.

The ingrowth of excess ^231^Pa and ^230^Th, generated by the decay of ^235^U and ^234^U in the overlying water column (xs) at the time of deposition (Paxs,0 and Thxs,0, where 0 indicates the decay corrected values), has been calculated from the measured bulk concentrations^22^. Lithogenic contributions were corrected by applying a detrital ^238^U/^232^Th activity ratio of 0.5 based on a basin-wide average lithogenic background as suggested by Henderson and Anderson^22^ or Bourne et al.^62^. As well, a disequilibrium of 4% for ^234^U/^238^U in the lithogenic fraction has been considered to account for preferential ^234^U loss via the recoil-effect^62^.

We excluded samples SO213-76-2 200 cm and 314 cm from our interpretations. These samples contain high concentrations of volcanic glass that may have interfered with our measurements. Nevertheless, all measurements, including SO213-76-2 200 cm and 314 cm, are included in the PANGAEA datafiles (https://doi.pangaea.de/10.1594/PANGAEA.889934).

#### Opal measurements

As the presence of biogenic opal may affect the ratio of ^231^Pa and ^230^Th, by preferentially scavenging ^231^Pa^Ref.^^25^, we determined the sedimentary biogenic opal content of our samples following the method of Müller and Schneider^63^. For the analysis, we leached 20–100 mg of bulk sediment, using 100 ml of 1.0 M NaOH at 85 °C. The alkaline solution was then injected into the analyzer. In the analyzer, the leachate was treated with 0.088 M H_2_SO_4_, a molybdate reagent, oxalic acid and an ascorbic acid in order to form molybdate-blue complexes, which were analyzed using a photometer for dissolved silica. For most sediment cores, the average SiO_2_ content was well below 2%. Since these low biogenic opal concentrations would only marginally affect the sedimentary ^231^Pa/^230^Th-ratios^26,64,65^, and since the signals of both metrics did not covary, we dismiss any significant impact of biogenic opal in driving downcore changes in ^231^Pa/^230^Th. Rather, we interpret changes in sedimentary ^231^Pa/^230^Th as primarily reflecting temporal changes in overturning circulation.

#### Biogenic barium

Biogenic barium (Ba_Bio_) is considered to be a reliable proxy for paleoproductivity^66^. Ba_Bio_ was calculated based on the difference of total Ba and lithogenic Ba (Ba_lith_), measured on our bulk samples via ICP-MS. To calculate Ba_lith_ from ^232^Th (measured by ICP-MS via isotope dilution), we followed the method of Costa et al.^67^: Ba_lith_ = 51.4 * ^232^Th.

To assess the role, vertical mass fluxes and paleoproductivity might have played on the ^231^Pa/^230^Th-ratios in the Southwest Pacific, we compared both, Th_xs0_ (Fig. S5A) and Ba_Bio_ (Fig. S5B) to our ^231^Pa/^230^Th-records. Due to the weak correlation of these proxies, we are confident to interpret ^231^Pa/^230^Th in terms of paleocirculation (Figure S6).

## Supplementary Information

Supplementary Information.
