# Supplementary material for: Deglacial patterns of South Pacific overturning inferred from 231Pa and 230Th
Source: Sci Rep. 2021 Oct 14;11:20473. doi: 10.1038/s41598-021-00111-1 (PMC8517020; doi:10.1038/s41598-021-00111-1)
Supplement: Supplementary file 1 — Supplementary Information. [file 41598_2021_111_MOESM1_ESM.docx]

Supplementary material for:

**Deglacial patterns of South Pacific Overturning inferred from ^231^Pa/^230^Th**

Thomas A. Ronge, Jörg Lippold, Walter Geibert, Samuel L. Jaccard, Sebastian Mieruch-Schnülle, Finn Süfke, Ralf Tiedemann

**Supplementary text**

**Impact of biogenic opal**

Unlike the deep-water sediment cores from ~2000 m to ~4300 m (SO213-82-1 down to SO213-76-2), PS75/104-1 (835 m), which is not discussed in the main article, is bathed by newly formed Antarctic Intermediate Water (AAIW) and thus records waters that recently passed an area of high silicate production^1^. The noticeable decrease in the ^231^Pa/^230^Th-ratio of PS75/104-1 at ~20 ka might therefore not only record changes in circulation strength, but also to a certain extent enhanced ^231^Pa-scavenging in the opal-rich waters of AAIW-formation. This process would lower the ^231^Pa/^230^Th-ratio^2^ and thus might erroneously indicate enhanced overturning once the waters reach our core location. South of 45° S, in the area covered by our New Zealand Margin sediment cores, pCFC-data (partial pressure of chlorofluorocarbon-11) indicate an average age of Southwest Pacific AAIW of just 21-22 years^3^. If AAIW ages were similar over the past, this travel time would be too short to reset the ^231^Pa/^230^Th-signal. Hence, we assume that the signal recorded by PS75/104-1 also reflects the pattern of opal productivity in the formation area of Southwest Pacific AAIW, instead of being a pure circulation signal. This reasoning is corroborated by opal flux rates and ^231^Pa/^230^Th-ratios from the Southern Ocean, south of the Antarctic Polar Front^4^, which indicate the most pronounced increase in opal flux, parallel to the observed decrease in PS75/104-1 ^231^Pa/^230^Th-ratios (~17.2 – 15.5 ka). Hence, although the opal-values of PS75/104-1 are constantly below 1% (*https://doi.pangaea.de/10.1594/PANGAEA.889934*), we argue that the upstream opal-production affected the AAIW ^231^Pa/^230^Th-signal at our core site.

East Pacific Rise (EPR) record PS75/059-2 on the other hand shows higher glacial opal concentrations (~10%) compared to low Deglacial and Holocene values (3-5%). We assume that the glacial northward displacement of the Antarctic Polar Front (APF)5, shifted the area of higher opal production into the vicinity of PS75/059-2. During Termination 1 and the Holocene, the southward movement of the APF led to lower opal concentrations. Hence, we assume that after ~18 ka, ^231^Pa/^230^Th in PS75/059-2 was not driven by opal concentrations in the first order but can act as an overturning proxy for deep-water at the EPR.

We see no significant impact of opal on the NZM sediment cores between 2066 m and 4339 m water depth. Hence, we are confident to use the ^231^Pa^/230^Th-proxy to reconstruct Southwest Pacific overturning.

***Hummingage* age-depth model**

In its current version, *hummingage* requires Gaussian errors of the input age data, which is a fair assumption on our already calibrated data. *Hummingage* applies a multi-segment linear regression on the accumulation rate of the sediment data. The decision on the optimal number of segments is made with the objective support of the BIC (Bayesian Information Criterion)^6^. The BIC implements the concept of Occams’s Razor^7^, which suggest choosing the hypothesis requiring the fewest assumptions among competing hypotheses describing a phenomenon (equally) well. Practically the BIC is an optimal compromise between minimizing the fit residuals and dividing the data into not more segments as needed. For our data, the BIC suggested to divide the sequence in either two or three segments.

Following the linear regression on the accumulation rate *hummingage* applies a Bayesian tuning to find a best fitting curve by interpreting the regression as prior probability and ask for the posterior probability of this regression given the observed data. Following a Gaussian assumption, it turned out that the best fitting curve is the weighted average of the regression line and the original data, whereas the weights are given by the regression error and the individual data errors.

Because of the novelty of the *hummingage* method we also applied the widely used *Bacon* method to our data (Fig. S2). Since our data have been already calibrated, we disabled the calibration function in *Bacon* (cc=0). *Bacon* also divides the original data into segments and for our data it suggests roughly to use segments in the order of 10. Following, it is the subjective decision of the user, how many segments to choose until the curve “looks good”. One guideline to choose the best number of segments is to assure that the MCMC algorithm used by *Bacon* mixes well and finally, we decided to use 10, 11 or 12 segments. Lower numbers of segments show bad MCMC mixing and higher numbers of segments introduce unrealistic curvatures.

As can be seen from Figure S2, both the *hummingage* model and the *Bacon* model yield very similar results, especially also the errors are quite similar. This is an important point, because according to Trachsel et al.^8^, the strength of the Bayesian age-depth models, in comparison with the standard methods, is a better representation of the errors and thus a more reliable estimation of the age-depth relation. Thus, in our case, with the pre-calibrated data, *hummingage* is a real alternative, is easy to use online, and provides similar results to the sophisticated *Bacon* model. Finally, we decided to use the interpolated (1 cm) model output from *hummingage* for our analysis.

**Supplementary figures and tables**

**
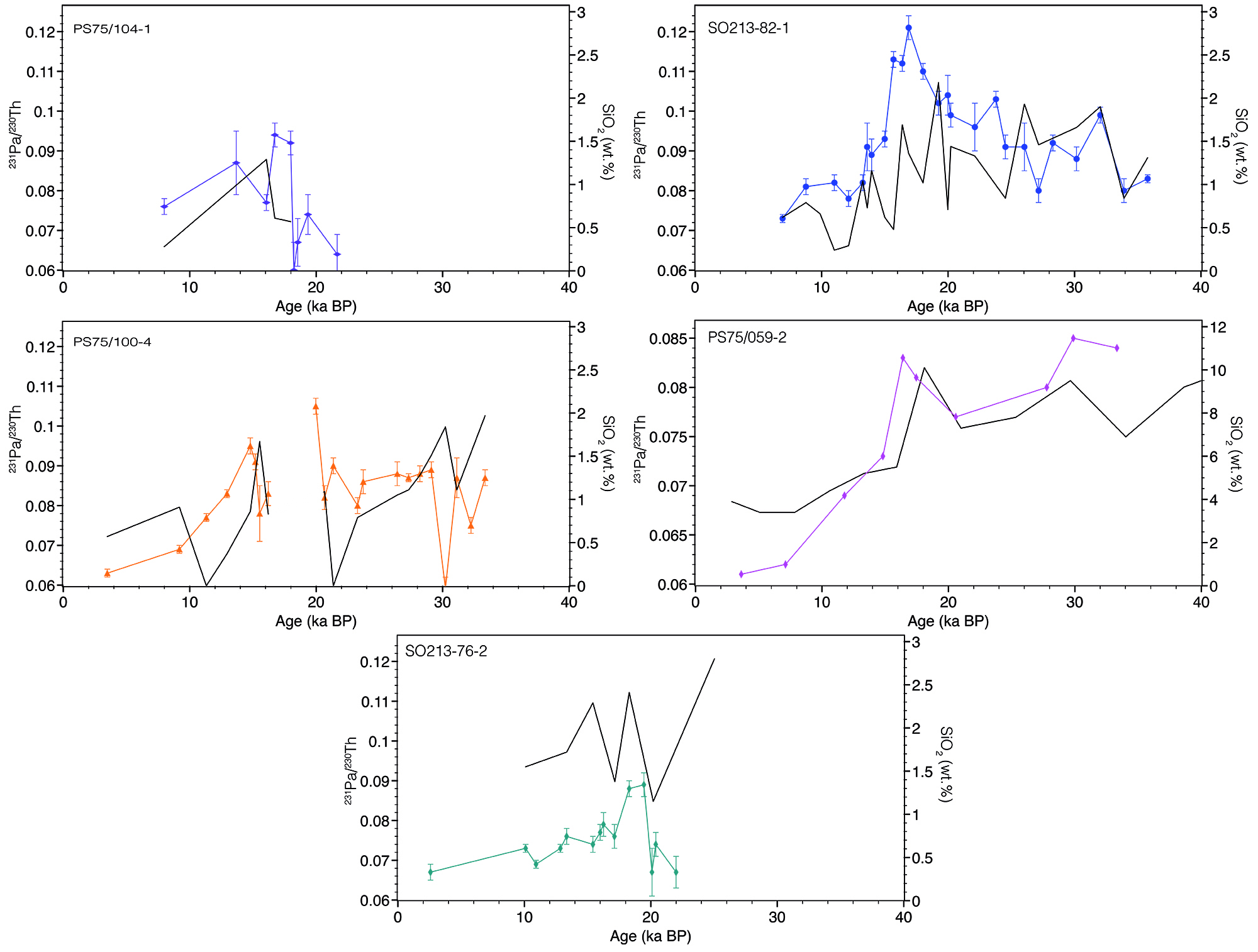
**

**Figure S1:** Downcore ^231^Pa/^230^Th records (colored) vs. SiO_2_ (black). Note the change of scale in the y-axis for the opal content for the Southern Ocean core PS75/059-2.


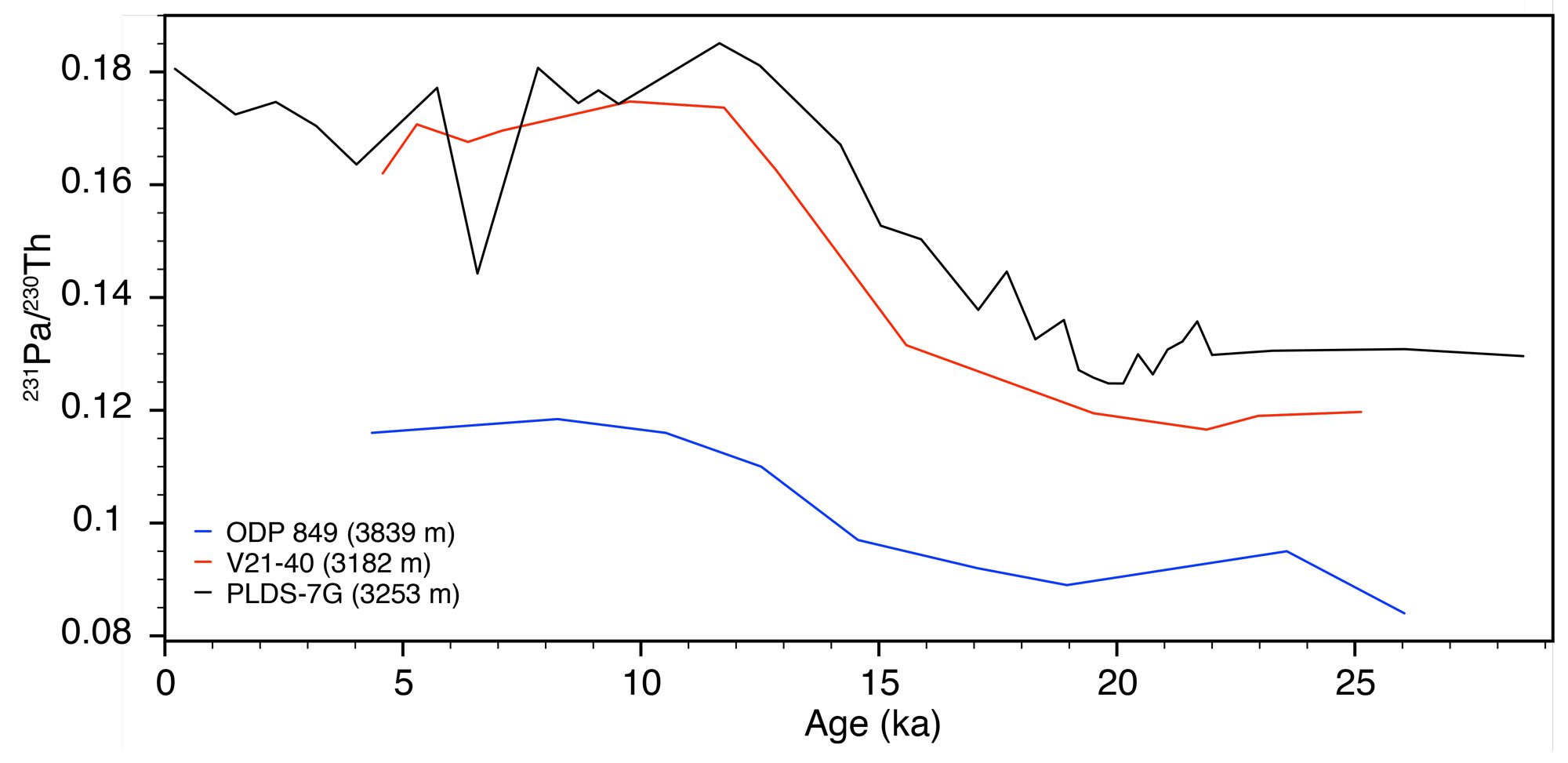


**Figure S2:** Records of Equatorial Pacific ^231^Pa/^230^Th along the East Pacific Rise^23-25.^


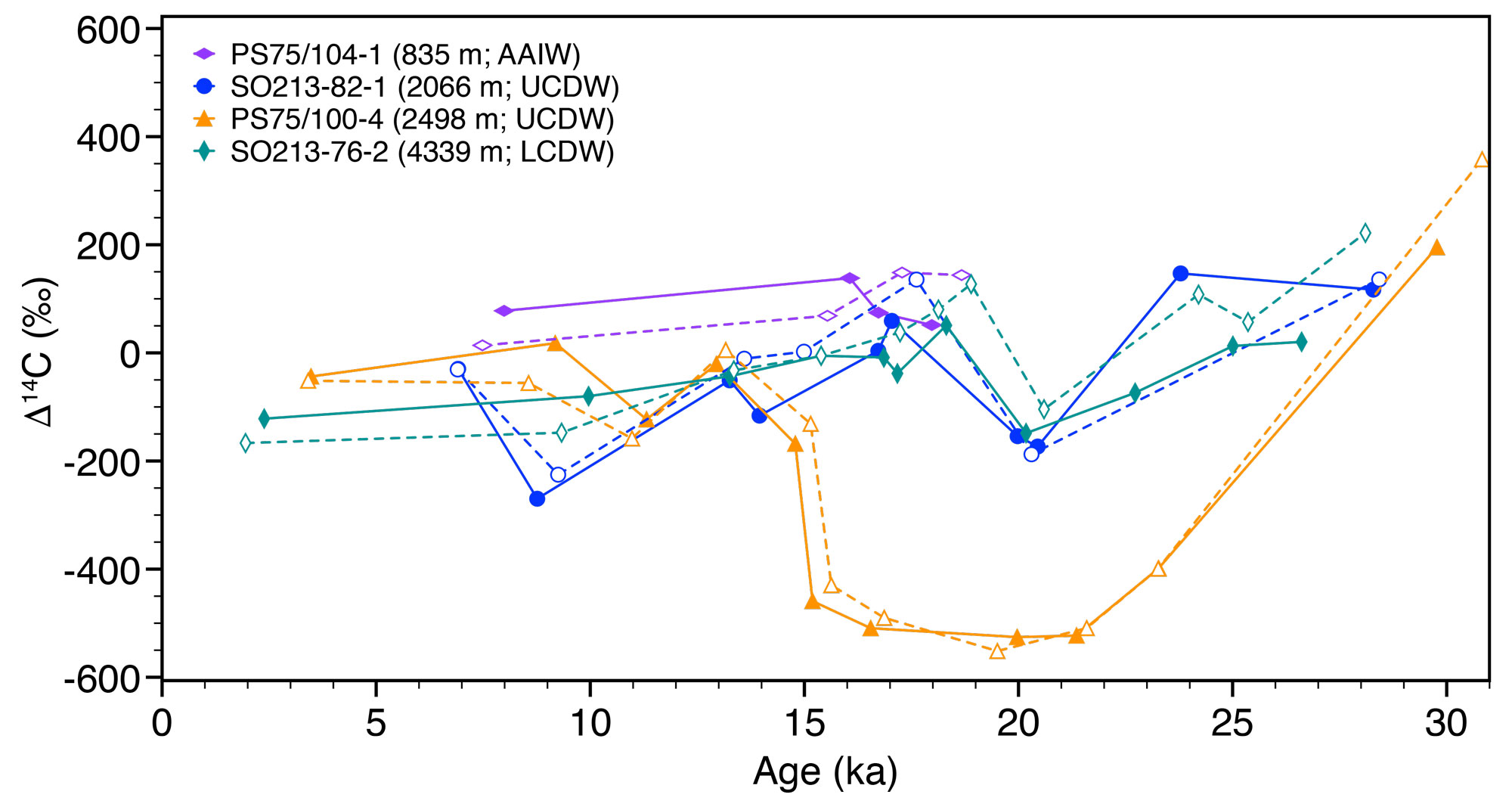


**Figure S3:** Comparison of Δ^14^C-records based on the Ronge et al.^9^ age models (broken lines) and our updated ^14^C-based age models (complete lines).


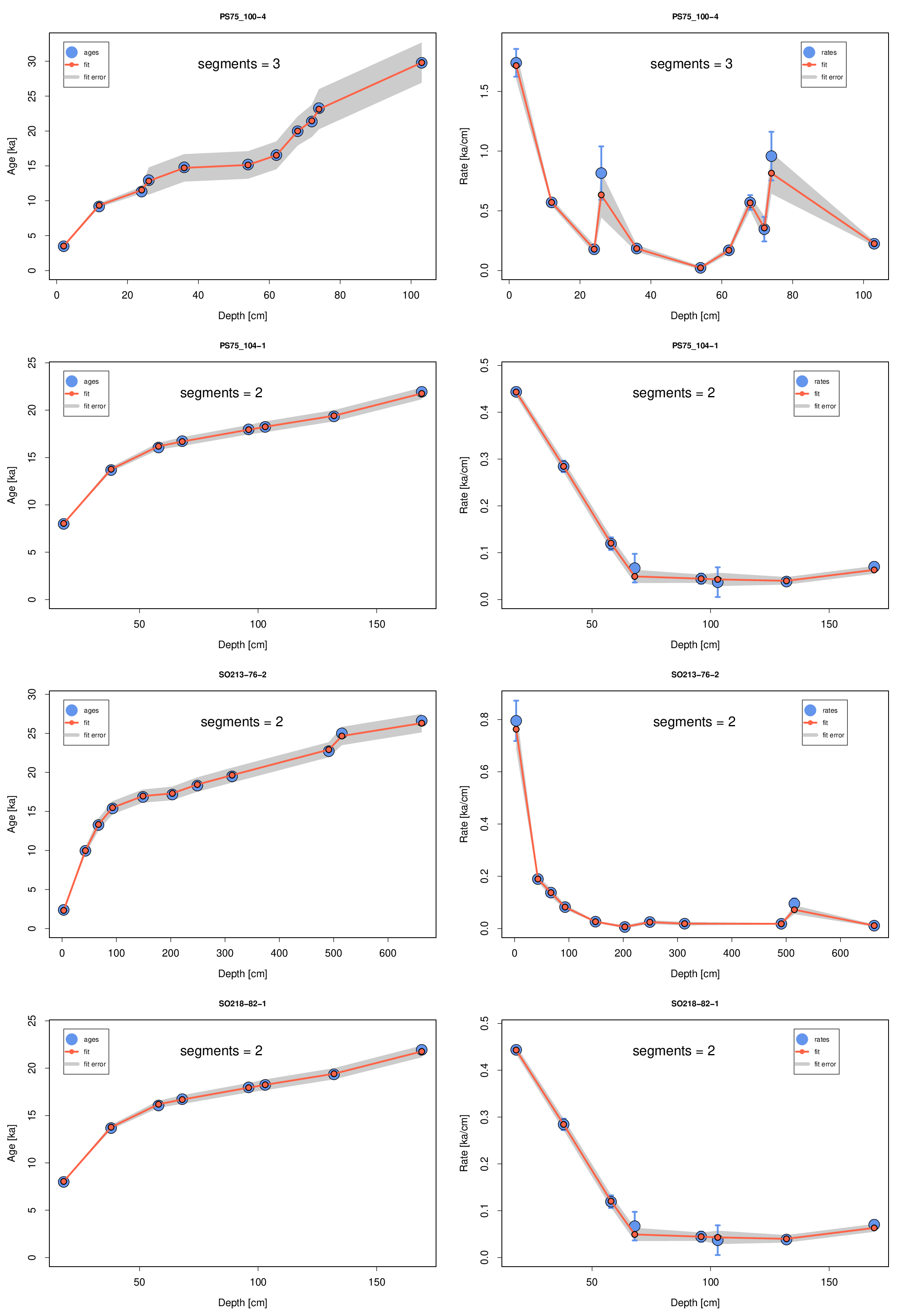

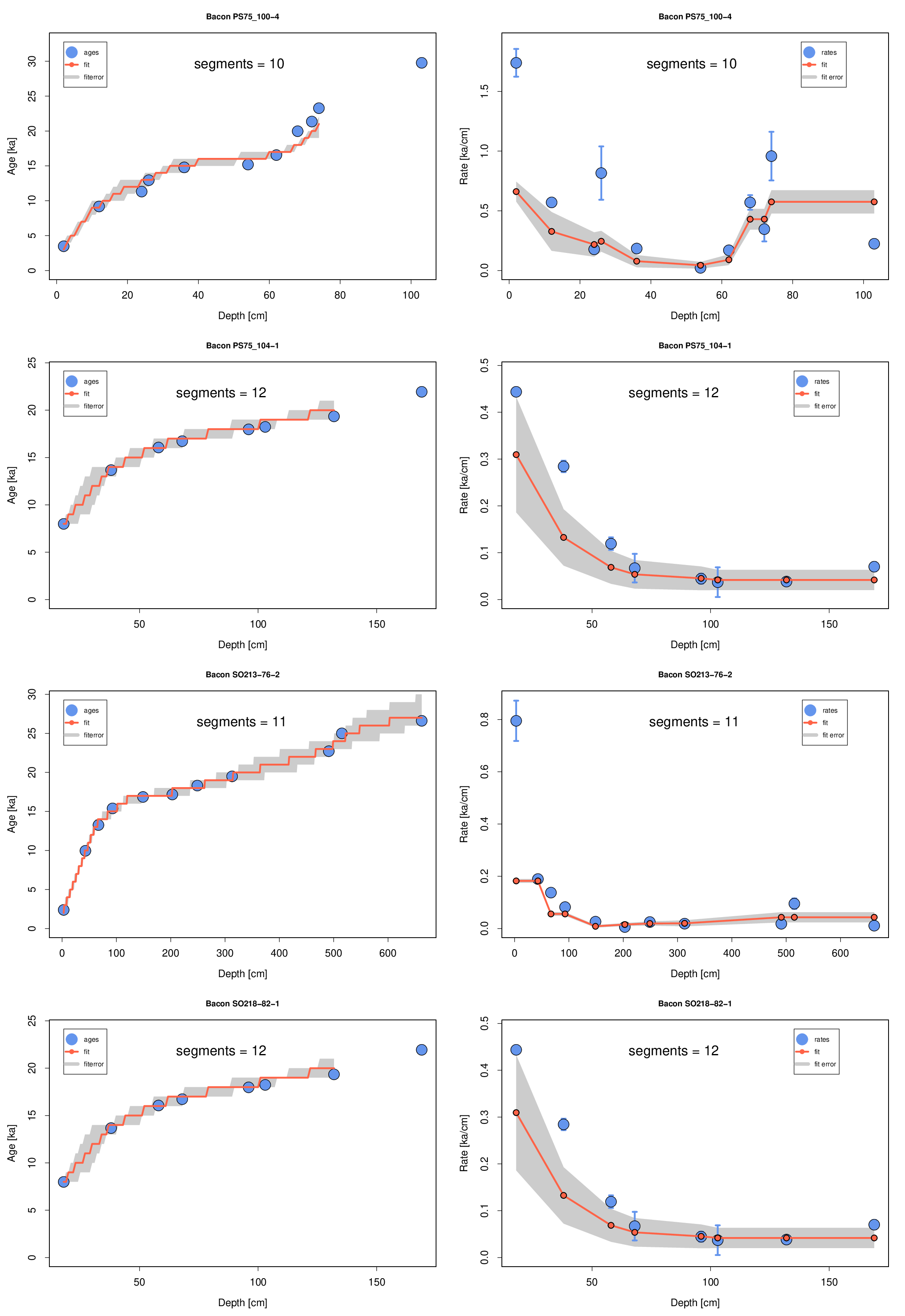


**Figure S4:** Bayesian approach to estimate the quality of our age models, using our *hummingage* (left) and the established *Bacon-*script (right).

**
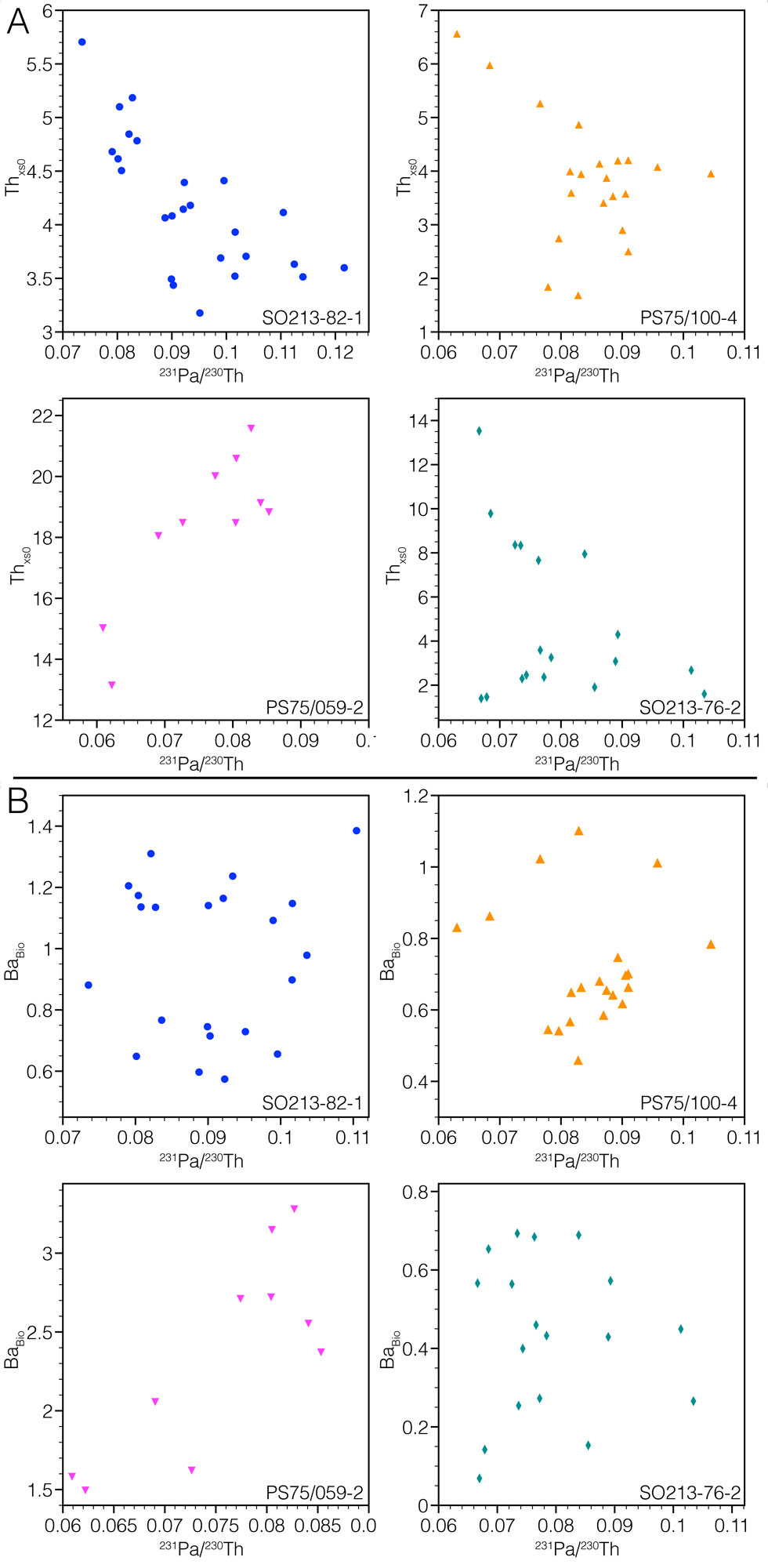
**

**Figure S5:** Comparison of ^231^Pa/^230^Th to Th_xso_ **(A)** and Ba_Bio_ **(B)** for all deep-water sediment records, measured on the same samples


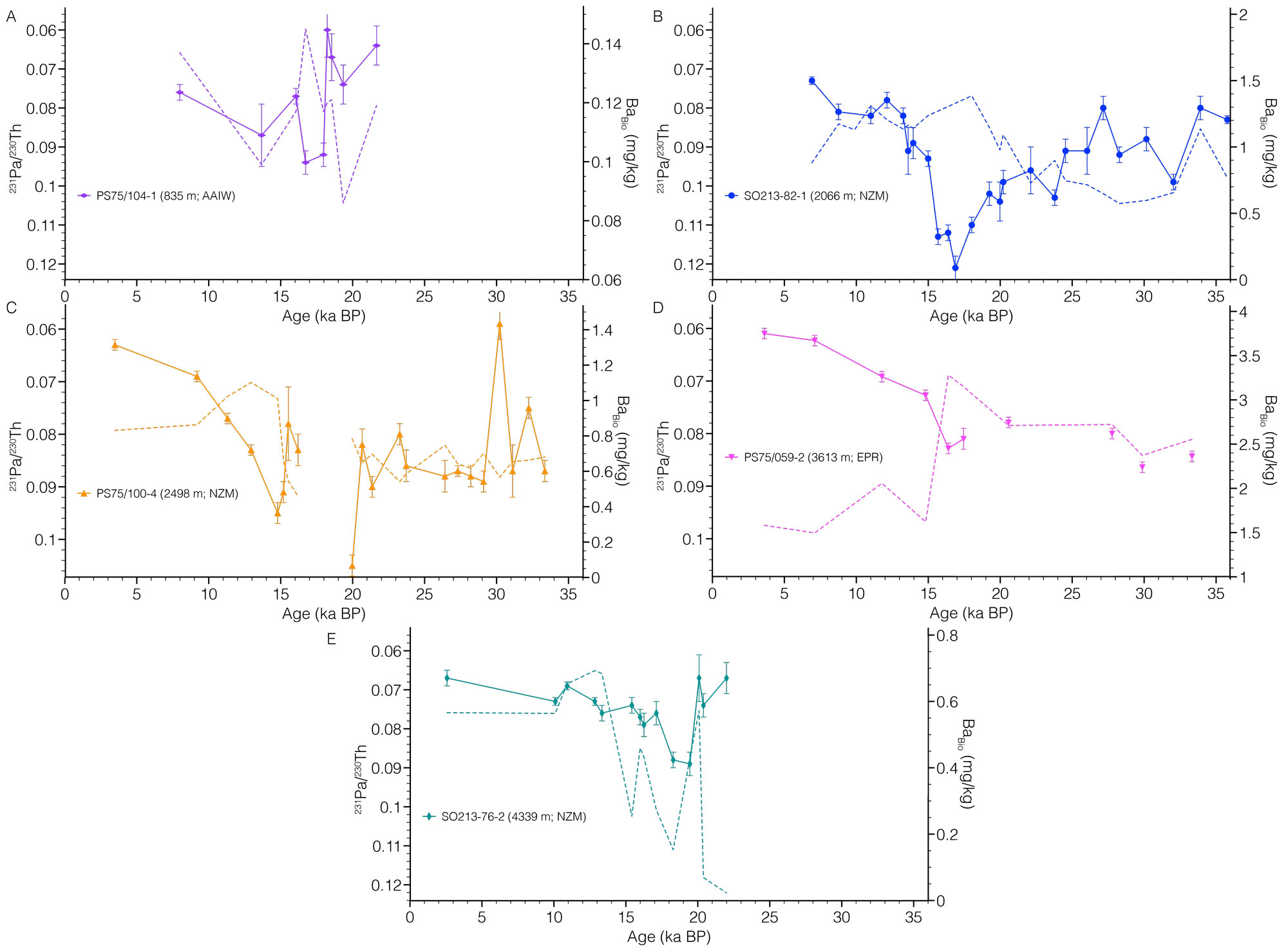


**Figure S6:** Comparison of ^231^Pa/^230^Th and Ba_Bio_ for all sediment records, measured on the same samples.

| **Station** | **Lat** | **Long** | **^231^Pa/^230^Th** | **Depth (m)** | **Reference** |
| --- | --- | --- | --- | --- | --- |
| V27-263 | 35.02 | -40.92 | 0.037 | 3704 | 10 |
| KNR140 31GGC | 30.90 | -74.50 | 0.066 | 3410 | 10 |
| RC13-189 | 1.86 | -30.00 | 0.056 | 3233 | 11 |
| V30-40 | -0.20 | -23.15 | 0.044 | 3706 | 11 |
| RC16-66 | -0.76 | -36.62 | 0.041 | 4424 | 11 |
| MD09-3257 | -4.24 | -36.35 | 0.067 | 2344 | 12 |
| MD95-2037 | 37.10 | -32.00 | 0.096 | 2159 | 13 |
| KN140-2-51GGC | 32.78 | -76.28 | 0.068 | 1790 | 14 |
| ODP 1063 | 33.69 | -57.61 | 0.057 | 4584 | 15 |
| 12JPC | 29.75 | -72.90 | 0.059 | 4250 | 16 |
| M35003 | 12.09 | -61.24 | 0.109 | 1300 | 16 |
| IODP 1313 | 41.00 | -32.96 | 0.051 | 3414 | 16 |
| ODP 1055 | 32.78 | -76.29 | 0.065 | 1798 | 17 |
| 55GGC | 4.90 | -42.90 | 0.045 | 4556 | 17 |
| 58GGC | 4.80 | -43.00 | 0.045 | 4341 | 17 |
| 71GGC | 4.40 | -43.70 | 0.059 | 3164 | 17 |
| 82GGC | 4.30 | -43.50 | 0.051 | 2816 | 17 |
| C2 PC-2121009 | -24.30 | -43.20 | 0.098 | 781 | 17 |
| MD3253 | -2.35 | -35.45 | 0.042 | 3867 | 18 |
| MD3254 | -2.80 | -35.42 | 0.042 | 3715 | 18 |
| MD09-3256Q | -3.55 | -35.38 | 0.047 | 3537 | 18 |
| MD3242 | -4.22 | -37.83 | 0.090 | 1008 | 18 |
| GeoB3936-1 | 12.72 | -59.00 | 0.078 | 1854 | 18 |
| GeoB3935-2 | 12.61 | -59.39 | 0.082 | 1558 | 18 |
| GeoB3937-2 | 12.56 | -58.77 | 0.079 | 1654 | 18 |
| OCE326-GGC5 | 33.70 | -57.60 | 0.056 | 4550 | 19 |
| GeoB16202-2 | -1.91 | -41.59 | 0.051 | 2248 | 20 |
| JC094-GVY14 | 15.46 | -50.99 | 0.065 | 2714 | 21 |
| EW9209 1JPC | 5.91 | -44.20 | 0.043 | 4056 | 21 |
| ODP 1059 | 31.67 | -75.42 | 0.061 | 2985 | 22 |
| ODP 1060 | 30.76 | -74.57 | 0.054 | 3481 | 22 |
| ODP 1061 | 29.98 | -73.60 | 0.057 | 4038 | 22 |
| ODP 1062 | 28.25 | -74.41 | 0.052 | 4761 | 22 |
| GeoB1515 | 4.24 | -43.67 | 0.051 | 3129 | 22 |
| GeoB1523 | 3.83 | -41.62 | 0.062 | 3292 | 22 |

**Table S1:** Sample details and references for W-Atlantic data used in Figure 1.


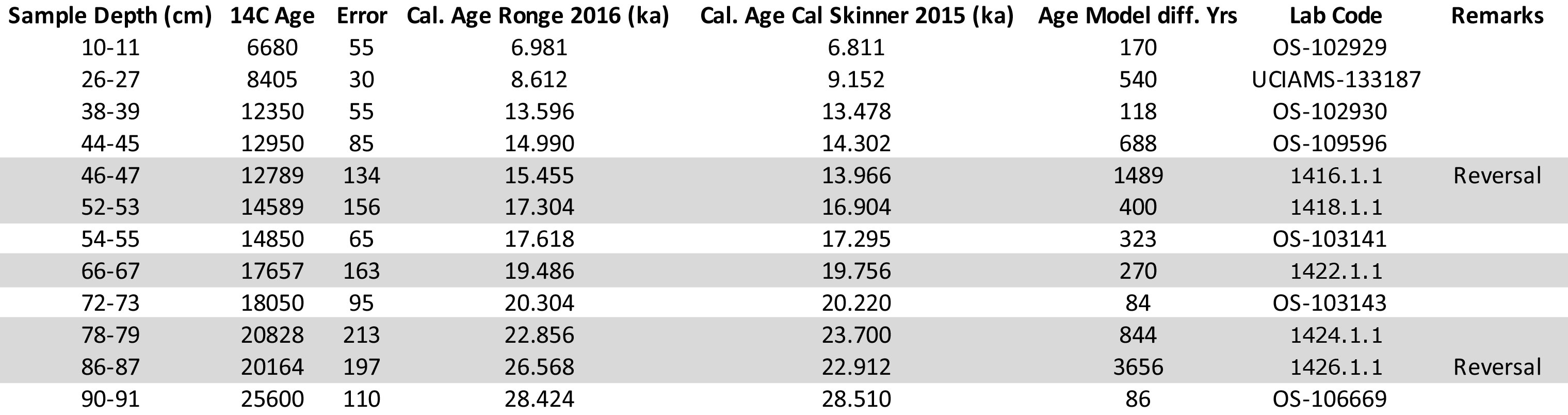


**Table S2:** Radiocarbon ages for sediment core SO213-82-1. Samples marked by grey shading indicate new measurements conducted for this study. Other samples were previously measured by Ronge et al.^9^. Cal. Age Ronge (2016) indicates calendar ages according to the ^14^C-independent method by Ronge et al. ^9^. Cal. Age Skinner 2015 is the ^14^C-derived age model, with surface reservoir ages accounted for according to Skinner et al.^26^. Age Model diff. is the difference in years between both methods.

Supplementary References:

1 Bostock, H. C., Sutton, P. J., Williams, M. J. M. & Opdyke, B. N. Reviewing the circulation and mixing of Antarctic Intermediate Water in the South Pacific using evidence from geochemical tracers and Argo float trajectories. *Deep-Sea Research I* **73**, 84-98 (2013).

2 Rutgers van der Loeff, M. *et al.* Meridional circulation across the Antarctic Circumpolar Current serves as a double ^231^Pa and ^230^Th trap. *Earth and Planetary Science Letters* **455**, 73-84 (2016).

3 Rintoul, S. R. & Bullister, J. L. A late winter hydrographic section from Tasmania to Antarctica. *Deep-Sea Research I* **46**, 1417-1454 (1999).

4 Anderson, R. F. *et al.* Wind-Driven Upwelling in the Southern Ocean and the Deglacial Rise in Atmospheric CO_2_. *Science* **323**, 1443-1448 (2009).

5 Benz, V., Esper, O., Gersonde, R., Lamy, F. & Tiedemann, R. Last Glacial Maximum sea surface temperature and sea-ice extent in the Pacific sector of the Southern Ocean. *Quaternary Science Reviews* **146**, 216-237 (2016).

6 Schwarz, G. Estimating the Dimension of a Model. *Annals of statistics* **6**, 461-464 (1978).

7 Sivia, D. S. & Skilling, J. *Data Analysis: A Bayesian Tutorial*. (Oxford University Press, 2006).

8 Trachsel, M. & Telford, R. J. All age-depth models are wrong, but are getting better. *The Holocene* **27**, 860-869 (2017).

9 Ronge, T. A. *et al.* Radiocarbon constraints on the extent and evolution of the South Pacific glacial carbon pool. *Nature Communications* **7:11487** (2016).

10 Bradtmiller, L. I., Anderson, R. F., Fleisher, M. & Burckle, L. H. Opal burial in the equatorial Atlantic Ocean over the last 30 kyr: implications for glacial-interglacial changes in the ocean silicon cycle. *Paleoceanography* **22**, PA4216 (2007).

11 Bradtmiller, L. I., McManus, J. & Robinson, L. F. ^231^Pa/^230^Th evidence for a weakened but persistent Atlantic meridional overturning circulation during Heinrich Stadial 1. *Nature Communications* **5**, 5817 (2014).

12 Burckel, P. *et al.* Atlantic Ocean circulation changes preceded millennial tropical South America rainfall events during the last glacial. *Geophysical Research Letters* **42**, 2014GL062512 (2015).

13 Gherardi, J.-M. *et al.* Glacial-interglacial circulation changes inferred from ^231^Pa/^230^Th sedimentary record in the North Atlantic region. *Paleoceanography* **24**, PA2204 (2009).

14 Hoffmann, S. S., McManus, J. & Swank, E. Evidence for Stable Holocene Basin-Scale Overturning Circulation Despite Variable Currents Along the Deep Western Boundary of the North Atlantic Ocean. *Geophysical Research Letters* **45**, 13427-13436 (2018).

15 Lippold, J., Gherardi, J.-M. & Luo, Y. Testing the ^231^Pa/^230^Th paleocirculation proxy - A data versus 2D model comparison. *Geophysical Research Letters* **38**, L20603 (2011).

16 Lippold, J. *et al.* Deep water provenance and dynamics of the (de)glacial Atlantic meridional overturning circulation. *Earth and Planetary Science Letters* **445**, 68-78 (2016).

17 Lippold, J. *et al.* Strength and geometry of the glacial Atlantic Meridional Overturning Circulation. *Nature Geoscience* **5**, 813-816 (2012).

18 Lippold, J. *et al.* Constraining the variability of the Atlantic Meridional Overturning Circulation during the Holocene. *Geophysical Research Letters* **46**, 11338-11346 (2019).

19 McManus, J. F., Francois, R., Gherardi, J.-M., Keigwin, L. D. & Brown-Leger, S. Collapse and rapid resumption of Atlantic meridional circulation linked to deglacial climate changes. *Nature* **428**, 834-837 (2004).

20 Mulitza, S. *et al.* Synchronous and proportional deglacial changes in Atlantic meridional overturning and northeast Brazilian precipitation. *Paleoceanography* **32**, 622-633 (2017).

21 Ng, H. C. *et al.* Coherent deglacial changes in western Atlantic Ocean circulation. *Nature Communications* **9**, 2947 (2018).

22 Süfke, F. *et al.* Constraints on the northwestern Atlantic deep water circulation from ^231^Pa/^230^Th during the last 30,000 years. *Paleoceanography and Paleoclimatology* **34**, 1945-1958 (2019).

23 Pichat, S. et al. Lower export production during glacial periods in the equatorial Pacific derived from 231Pa/230ThXS,0 measurements in deep-sea sediments. Paleoceanography 19, PA4023 (2004).

24 Bradtmiller, L. I., Anderson, R. F., Fleisher, M. & Burckle, L. H. Diatom productivity in the equatorial Pacific Ocean from the last glacial period to the present: A test of the silicic acid leakage hypothesis. Paleoceanography 21, 2006PA001282 (2006).

25 Thiagarajan, N. & McManus, J. Productivity and sediment focusing in the Eastern Equatorial Pacific during the last 30,000 years. Deep-Sea Research I 147, 100-110 (2019).

26 Skinner, L. C. et al. Reduced ventilation and enhanced magnitude of the deep Pacific carbon pool during the last glacial period. Earth and Planetary Science Letters 411, 45-52 (2015).
